# First Isolation and Molecular Typing of Pathogenic and Intermediate *Leptospira* Species from Urine of Symptomatic Dogs

**DOI:** 10.3390/vetsci8120304

**Published:** 2021-12-02

**Authors:** Ivana Piredda, Loris Bertoldi, Giuseppe Benvenuto, Bruna Palmas, Aureliana Pedditzi, Pierangela Pintore, Valentina Chisu

**Affiliations:** 1 Laboratory of Seroimmunology, Animal Health Department, Istituto Zooprofilattico Sperimentale della Sardegna, Via Vienna 2, 07100 Sassari, Italy; bruna.palmas@izs-sardegna.it (B.P.); aureliana.pedditzi@izs-sardegna.it (A.P.); pierangela.pintore@izs-sardegna.it (P.P.); valentina.chisu@izs-sardegna.it (V.C.); 2 BMR Genomics s.r.l., 35131 Padova, Italy; loris.bertoldi@bmr-genomics.it (L.B.); giuseppe.benvenuto@bmr-genomics.it (G.B.)

**Keywords:** leptospirosis, intermediate *Leptospira hovindhougenii*, dogs, MLST

## Abstract

Aim of this study was to evaluate, the presence and diversity of *Leptospira* spp. in blood and urine samples collected from 175 owned-dogs from Sardinia, Italy. After determination of leptospiral infection by microscopic agglutination test (MAT), urine from MAT-positive dogs were examined by real-time polymerase chain reaction (*lipL32* rt-PCR) and then isolated by culture. In order to characterize obtained serovars, positive cultures were then subjected to *16S* rRNA and *secY* sequencing, phylogenetic analysis and Multilocus Sequence Typing (MLST). Results showed that seven dogs (4%; 95% CI: 0–55) had *Leptospira* DNAs in their urine and five strains were isolated from urine cultures. The three different sequence types (ST17, ST198 and ST24) belonging to *Leptospira interrogans* genomospecies identified by MLST analyses in this study, confirmed that the leptospiral infection was widespread in Sardinian dogs. We also reported the first characterization of a new *Leptospira* spp. isolated from urine of one dog living in the study area. Whole genome sequencing and phylogenetic analysis, confirmed that this genospecies was closely related to *Leptospira hovindhougenii*, an intermediate *Leptospira* spp. with unknown pathogenicity previously isolated from a rat in Denmark. Further studies are required to clarify whether healthy dogs that shed leptospires in their urine could represent a zoonotic risk for humans in this region.

## 1. Introduction

Leptospirosis is a globally distributed zoonotic disease caused by infection with bacterial pathogens of the genus *Leptospira* [1]. Thus far, over 300 serovars of pathogenic *Leptospira* species have been described worldwide and many of which are well-established threats to domestic animals and livestock [2,3,4,5]. Although a wide range of *Leptospira* reservoirs have been described in the last few years, rats represent the commonly recognized reservoir of *Leptospira* species and the primary animal source of human outbreaks worldwide [6,7]. Rodent harbor leptospires in their proximal renal tubules and shed the spirochetes into the environment via urine [8,9]. Humans and domestic animals can become infected through direct contact with urine of infected animals or indirectly via the contaminated environments [10,11]. Leptospiral infection can manifest itself through a wide diversity of clinical manifestations that depend on both the virulence of the infecting strain and immunological status of infected hosts. In dogs, the severity of the disease is variable and the infection can range from mild and transient signs (such as isolated relapsing fever, anorexia, polydipsia and polyuria, lethargy, and hepato-splenomegaly), to more severe forms of the disease (including acute kidney injury, liver failure, meningitis, and respiratory distress) and death [2,12]. Since some infected dogs do not show any signs of illness, specific examination is crucial in this context, and an early diagnosis of leptospirosis could help readily initiate antibiotic therapy and decide the more effective treatment for the patient.

Regarding the high number of asymptomatic dogs, the true burden of the disease could be underestimated, and it can lead to neglecting the real rate of infection. The presence of pathogenic *Leptospira* spp. in domestic dogs has been well characterized, with serovars Canicola, and Icterohaemorrhagiae identified as the major serovars infecting dogs worldwide [13,14,15,16]. In Europe, dog vaccination against these serovars is used since the 1960s [17]. More recently, the rapid widespread of Grippotyphosa and Bratislava serovars in Europe allowed to reexamine the existing vaccine and to formulate a vaccine in which the new serovars have been added and licensed in European countries (Licence number EU/2/12/143/001-004). In Sardinia, current literature still lacks information on the presence and typing of *Leptospira* species in dogs. Only one study reports the presence of different serovars belonging to pathogenic *Leptospira* serogroups isolated from asymptomatic dogs living in the island [18].

In order to improve the scientific knowledge about *Leptospira* serovars circulating in Sardinian dogs, this study aimed to: (a) identify canine *Leptospira* serovars by serological and molecular techniques, (b) isolate *Leptospira* strains from urine samples of dogs that resulted positive after serological exams, (c) characterize the new *Leptospira* serovars by phylogenetic analysis of the newly identified sequences.

## 2. Materials and Methods

### 2.1. Animal Ethics

This study adhered to strict guidelines outlined by the ethical committee of the Isti-tuto Zooprofilattico Sperimentale della Sardegna (IZS). In addition, permission was granted by the Italian Ministry of Health (Ministero della Salute) in accordance with Council Directive 2010/63/EEC of the European Union and the Italian D.Lgs 26/2014 (protocol 1248/2015-PR) whose representatives personally oversaw that animals were handled with respect according to the laws on experimental animal care. Written informed consent was also obtained from each dog owner prior to sample collection.

### 2.2. Animals and Study Groups

**Figure 1:**
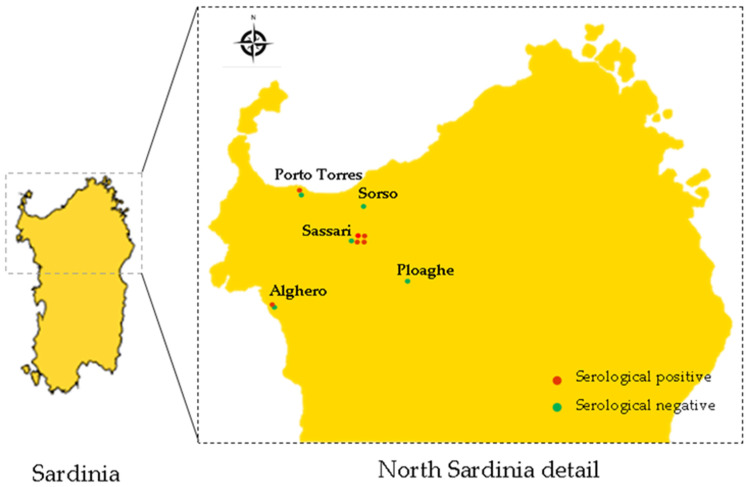
Map of Northern Sardinia indicating number and location of Veterinarian Clinics that adhered to this study. Red and green dots indicate Clinics in which dogs were found seropositive and seronegative, respectively.

Between January 2019 and December 2020, eleven Veterinary Clinics gave their consent to participate to this study and to collect urine and blood samples from each dog. Clinics were located near the Metropolitan City of Sassari in the North of Sardinia, Italy (Figure 1). A consent form was obtained from the dog owners to include their animals in this study. Dog owners of were also informed about the risk associated with collection of urine samples. Veterinarians performed clinical examination on each dog by evaluating health condition, degree of hydration, inspection of the skin and mucous membranes, palpation of lymph nodes and abdominal organs, cardiopulmonary auscultation, and rectal temperature measurement. Moreover, dog owners were interviewed to obtain information about place of origin, living environment (e.g., urban or rural setting or if dogs spent the night indoors or outdoors), history of vaccination and use of prophylactic measures to prevent infestations of wild rodents. The age and gender were also obtained from each dog (Table 1). About the age criteria, dogs were classified into two main groups: dogs under 2 years old and dogs over 2 years old (Table 1). On the basis of clinical manifestations, dogs were also divided in two groups: one group included dogs that were judged to be in good health, with no obvious clinical signs, while the other group included dogs that presented symptoms compatible with leptospiral infection (fever, anorexia, weight loss, prostration, vomiting, gingival lesions, jaundice, hemorrhagic disorders, hyperoxia).

### 2.3. Sample Collection

Blood samples were collected from cephalic vein and packed in tubes with EDTA anti-coagulant (Vacutainer sterile, R, IVD, Padova, Italy). After sampling, tubes were transferred to the sero-immunology laboratory of the Istituto Zooprofilattico Sperimentale of Sardinia (IZS) for further analyses. In order to detect possible signs related to a latent infection and to an increase in antibody titers (that could reach peak levels within two to three weeks after leptospiral infection) [19], the follow-up visit was done within 15 days from the first visit. During the following visits, dogs were submitted to the same clinical examination as above. Veterinarians collected urine specimens from each dog by catheterization or cystocentesis after administration of furosemide given by mouth in the form of tablets (1–2 mg/kg). Urine was placed in a sterile tube containing a culture medium for sample transport, named *Leptospira* Transport Medium (LTM), as described in Guedes et al. [20], and then stored at −20 °C until use.

Finally, two kidney samples were collected from two dogs that died of respiratory distress and severe hepato-nephritis, respectively, due to acute leptospirosis.

### 2.4. Microscopic Agglutination Test (MAT)

All serum samples were subjected to the microscopic agglutination test (MAT), test as previously described [18]. MAT was performed using a panel of 9 serovars comprising of leptospires from the Mediterranean area. Serovars used were: *L.* Sejroe Hardjo, *L.* Australis Bratislava, *L.* Grippotyphosa Grippotyphosa, *L.* Pomona Pomona, *L.* Tarassovi Tarassovi, *L.* Icterohaemorrhagiae Icterohaemorrhagiae, *L.* Icterohaemorrhagiae Copenhageni, *L.* Canicola Canicola, and *L.* Ballum Ballum. All sera were diluted with normal saline (pH 7.2–7.4) starting at 1:10 (WHO 2003). Diagnostic cultures were added to wells of polystyrene plates, then one drop of sample serum was added to the appropriate wells. The plates were covered to prevent evaporation and were incubated at 29 °C for 2 h. Pure saline solution was used as negative control. The analysis was performed in dark field microscopy (Olympus BX50; Olympus Corp., Tokyo, Japan) with magnification of 100×. Samples were considered positive when 50% or more leptospires were agglutinated at 10^−2^ dilution, considering a cut-off titer of ≥1:100. Samples that showed positive agglutination were subjected to serum titration to determine the titer.

Since dogs from this study were all immunized against *Leptospira* spp., and a previous vaccination could impact their antibody titers, samples having a MAT titer ≥1:400 were considered positive. In order to evaluate an active infection or a seroconversion, all dogs with titers ranging from 1:100 to 1:400 were followed up to monitor the dynamics of antibody titers against *Leptospira* spp.

### 2.5. Culture Conditions

Ellinghausen–McCullough–Johnson–Harris (EMJH) semi-solid and liquid medium was used for the isolation of *Leptospira* spp. from urine and dog kidneys. 5-Fluorouracil (5-FU) was added due to minimize bacterial contamination. Samples of kidney were homogenized as previously described [18]. A total of 1000 µL of urine and 25 mg of tissue were suspended in EMJH–fluorouracil semisolid medium at 28 °C and cultured for a period of three months. The media were examined under dark-field microscopy for the presence of leptospires approximately every seven days. Pure isolates, free of contaminants, were used for further molecular identification.

### 2.6. Molecular Detection of Leptospira spp. by Multiplex qPCR

Urine samples and isolates were extracted using the DNeasy Blood and Tissue Kit^®^ (Qiagen, Hilden, Germany), in according to the manufacturer’s instructions. Quantitative PCR (q-PCR) assay was performed by using primers LipL32-45F (5′-AAGCATTACCGCTTGTGGTG-3′), LipL32-286R (5′-GAACTCCCATTTCAGCGATT-3′), with the probe LipL32-189P (FAM-5′-AAAGCCAGGACAAGCGCCG-3′-BHQ1) [21], combined with primers Gender16S-P1 forward (5′-TAGTGAACGGGATTAGATAC-3′), and Gender16S-P2 reverse (5′-GGTCTACTTAATCCGTTAGG-3′), and probe Gender16S-Prob (Cy5-5′-AATCCACGCCCTAAACGTTGTCTAC-3′-BHQ2) [22] that amplified 242 and 104 bp of the *lipL32* and *16S* rRNA genes, respectively, due to detect *Leptospira* genomic DNA in urine, and kidneys of dogs from this study. A negative control (DNA extracted from water) and a positive control DNA extracted from the reference strain of *L. interrogans* (ATCC^®^ BAA1198D5TM) were included in each run. Samples were positive if the cyclic threshold (CT) values for the *lipL32* gene were comprised between 5 and 40 cycles. Samples were considered negative if no CT value, or a CT value ≥ 40, or a non-repeatable CT value was found.

### 2.7. Amplification of rrs and secY Genes

All five *Leptospira* isolates obtained (Table 2) were analyzed with a set of primers that amplified a fragment of 541 bp of the *16S* rRNA gene, and of 549 bp of the *secY* partial gene [23]. Negative and positive controls were included in each test (one positive and one negative control every 20 samples tested). The PCR reactions were performed by using a T100 Thermal Cycler (Bio-Rad apparatus, Milan, Italy). PCR products were visualized by electrophoresis in 1.5% agarose gel stained with SYBR-Safe DNA Gel Stain (Invitrogen, Carlsbad, CA, USA), and examined under UV transillumination.

### 2.8. Multi Locus Sequence Types (MLST)

In order to reveal the sequence types (STs) of *Leptospira* isolates, the MLST assay was performed by using the 7 housekeeping genes *pntA*, *sucA*, *tpiA*, *pfkB*, *mreA*, *glmU*, and *caiB* as previously proposed by Boonsilp et al. [24]. Each allele and the allelic profiles (glmU-pntA-sucA-tpiA-pfkB-mreA-caiB) were submitted to the *Leptospira* database (https://bigsdb.pasteur.fr/, accessed on 16 September 2021) to define the STs.

### 2.9. Purification, Sequencing, and Phylogenetic Analysis

Purified products were subjected to Sanger sequencing reactions using the BigDye™ Terminator v3.1 Cycle Sequencing Kit (Applied Biosystems, Monza, Italy), according to manufacturer specifications and specific primers. The readings were performed with Software that includes a normalization feature for use with the GeneScan™ 600 LIZ^®^ Size Standard v2.0 (GS600 LIZ v2—Applied Biosystems, Monza, Italy). Obtained sequences were analyzed and aligned with the reference sequence in the BLASTn database. For phylogenetic analysis, the 541 bp sequences obtained in this study were aligned with each other and with a set of 27 sequences representative of the *16S* rRNA gene variability of the different species belonging to *Leptospira.* The sequences were aligned with ClustalX [25]. Evolutionary analyses were conducted in MEGA 6 by using the Maximum Likelihood method based on the Kimura 2-parameter model. The bootstrap consensus tree inferred from 1000 replicates is taken to represent the evolutionary history of the taxa analyzed. The tree with the highest log likelihood (−1526.4247) is shown. The percentage of trees in which the associated taxa clustered together is shown next to the branches. Initial tree(s) for the heuristic search were obtained by applying the Neighbor-Joining method to a matrix of pairwise distances estimated using the Maximum Composite Likelihood (MCL) approach. A discrete Gamma distribution was used to model evolutionary rate differences among sites (five categories (+G, parameter = 0.3873)). The rate variation model allowed for some sites to be evolutionarily invariable ([+I], 36.5390% sites). The analysis involved 31 nucleotide sequences. All positions containing gaps and missing data were eliminated. There was a total of 475 positions in the final dataset.

### 2.10. Whole Genome Sequencing

The DNA library was prepared with the Illumina Nextera XT kit, following manufacturer instructions, then checked with BioAnalyzer (Agilent, Santa Clara, CA, USA) and quantified using Qubit fluorometer (Thermo Fisher, Bedford, MA, USA). Library was finally pooled with other samples, loaded on the MiSeq System (Illumina, Inc., Ann Arbor, MI, USA) and sequenced following the V3-300PE strategy. Raw reads were assessed for quality using FastQC (v0.11.7) (Illumina, Inc., Ann Arbor, MI, USA) and further processed by applying Cutadapt (v1.16) [26] to remove low quality bases (Q < 30), residual adapters and short sequences (length < 150). Cleaned reads were assembled with SPAdes (v3.12.0) [27] with standard parameters and assembly metrics calculated using QUAST (v4.C6.3) [28]. Finally, quantitative assessment of genome assembly was made with BUSCO (v5.1.2) [29] against spirochaetia_odb10 lineage. In parallel, Metaphlan 3 [30] was used to evaluate possible contaminants in the sequenced isolate and to characterize it at species level. To perform a phylogenetic analysis of the isolate, the assembled genome was com-pared with 13 *Leptospira* sequences downloaded from National Center for Biotechnology Information (NCBI, Bethesda, Rockville, ML, USA) using PhyloPhlAn 3 [31] in the accurate mode, with medium diversity parameter and choosing PhyloPhlAn database. The final refined tree was drawn with iTOL [32].

### 2.11. Statistical Analysis

Data obtained from seroprevalences and molecular diagnostic tests used for *Leptospira* spp. detection, were entered in a digital database. The description of all seropositive and positive samples was also performed. Standard deviation with the 95% confidence level was obtained for sample size calculation.

## 3. Results

### 3.1. Animal Classification and Clinical Symptoms

Among the 175 dogs analysed, 93 were male (53%; 95% CI: 49–57) and 82 were female (47%; 95% CI: 43–51). The age was known for all dogs. Specifically, 70 (40%; 95% CI: 36–44) dogs had an age between 1 month and 2 years and 105 (60%; 95% CI:56–64) were aged between 2 and 6 years (Table 1). Of the 175 tested dogs, 91% (160/175; 95% CI: 89–93) were vaccinated using a bivalent vaccine including serovars Icterohaemorrhagiae and Canicola from less than 6 months since the blood collection. In the 9% of dogs, blood sample was taken 6–12 months after Leptospira vaccination. Concerning the physical examination, dogs were also classified in two groups: healthy dogs (Nr. = 150) if no clinical abnormalities were detected and unhealthy dogs (Nr. = 25) if they showed clinical symptoms that consisted of increased body temperature, oral lesions, anorexia, vomiting, and icterus) relevant to leptospirosis. A nervous or behavioural disorders were also observed for each dog (Table 1).

Five symptomatic dogs needed hospitalization (dogs1–5 in Table 2). For three of them the infection resolved itself after antibiotic administration within one-three months and with no signs of sequelae. One dog (dog5) died after renal failure during the study, making it likely that they were previously infected with Leptospira. Dog2 was euthanized within 12 days after the onset of clinical disease due to severe renal failure.

**Figure 2:**
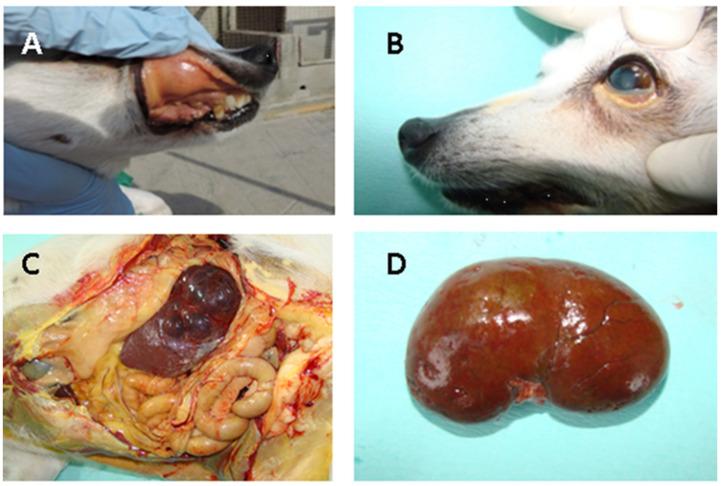
Acute lesions in dog5 naturally infected with *Leptospira* spp.: (**A**) Icterus of the oral conjunctival; (**B**) subcutaneous; (**C**) peritoneal cavity showing icterus of the visceral organs; (**D**) Diffusely congested kidney and spleen characterized by white spotting cortical lesions typical of leptospirosis.

Necropsy was allowed for both dogs, and macroscopic lesions of the kidneys were also documented. Congested kidney and spleen with white-spotted cortical lesions typical of leptospirosis in dog5 are illustrated in the Figure 2.

### 3.2. Microscopic Agglutination Test (MAT)

Out of 150 asymptomatic dogs, 12 (8%; 95% CI: 6–10) showed serum antibodies against *Leptospira* sorovar Bratislava (Nr. = 6), Icterohaemorrhagiae (Nr. = 4), Copenhageni (Nr. = 3), and Grippotyphosa (Nr. = 1). Titers ranged from 1:100 to 1:400 (data not shown). Out of the 25 symptomatic dogs, 5 (20%; 95% CI: 12–28) gave positive MAT results for one, three, or four different serovars. Specifically, most common serogroup found were Bratislava (Nr. = 5), Co-penhageni (Nr. = 4), Grippotyphosa (Nr. = 2), and Pomona (Nr. = 4). The information about MAT-positive dogs and the serum titers against serogroups used in this study have been shown in Table 2.

Three weeks from the first serum collection, all dogs with MAT titers ≥1:100 were re-tested and a titer modification was evaluated. Three dogs (dog1, dog3, and dog7) showed a threefold or greater rise in titer (Copenhageni 1:200 to 1:1600; Bratislava from 1:400 to 1:1600; Icterohaemorrhagiae 1:400 to 1:1600) which can be consistent with a recent infection. Six dogs exhibited decreasing titers and seven dogs showed the same titers or a slightly higher titer as before. Dog5 died before the second sampling was carried out (Table 3).

### 3.3. Molecular Results, Characterization and Sequencing of the Isolated Strains of Leptospira

Of the 17 dogs that tested positive after first MAT testing, seven (41%; 95% CI: 29–53), showed leptospiral DNA in their urine after rt-PCR amplification using the *16S* rRNA fragment (Table 2).

In total, 5 bacterial cultures were successfully isolated after approximately 30/40 days of incubation, one obtained from a kidney (14%; 95% CI: 1–27) and 4 from urine samples (57%; 95% CI: 38–76) (Table 4). The positivity of the isolates was confirmed by amplification of the *rrs* gene and further sequencing of the *secY* gene, which identified three different *Leptospira* strains named LEP_Seq-DOG1-4-5, LEP_Seq-DOG2, and LEP_Seq-DOG3. In particular, BLASTn analysis revealed that, the LEP_Seq-DOG1-4-5 sequences (derived from dog1, dog4 and dog5) obtained upon amplification of the *rrs* gene, showed 100% identity with pathogenic *L. interrogans*, while the sequences named LEP_Seq-DOG2 (derived from dog2) and LEP_Seq-DOG3 (from dog3) showed the highest homology with the intermediates *L. inadai* and *L. saintgironsiae* (100% identity), respectively. Sequencing of the *secY* amplicons obtained from dogs 1, 2, 4, and 5 revealed that the four sequences were 100% identical to those of the *L. interrogans* strains. MLST analysis of the five isolates yielded three different sequence types (ST), belonging to ST17 (derived from dog2 and dog4), ST198 (found in dog1), and ST24 (from dog5 urine). No amplification was obtained from dog3 after amplification of these genes. MLST results based on the seven-locus scheme obtained from *Leptospira* spp. isolates are reported in Table 4.

### 3.4. Phylogenetic Analysis Based on rrs Sequences

**Figure 3:**
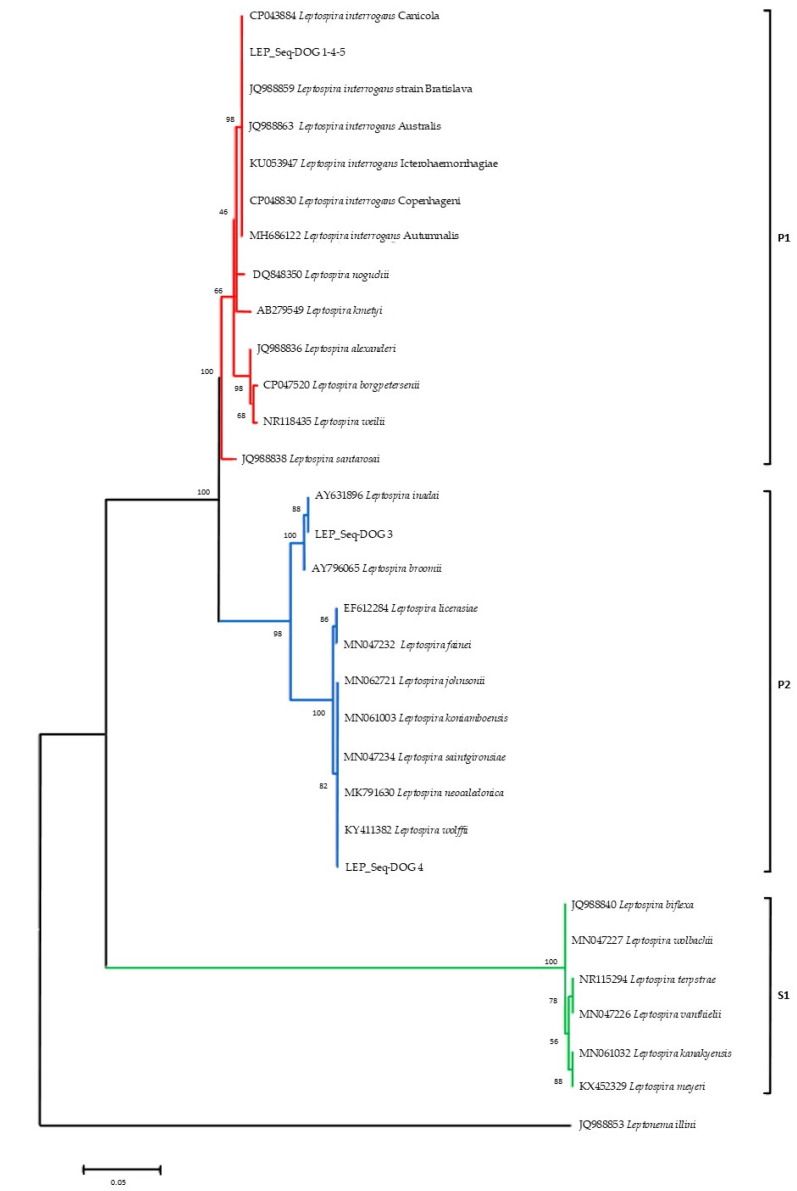
*16S* rRNA phylogeny of the identified *Leptospira* serovars and sequence representative of *Leptospira* species diversity. Pathogenic (P1), intermediate (P2), and saprophytic (S1) clades are indicated in red, blue, and green, respectively. Evolutionary analyses were conducted with MEGA 6 by using the Maximum Likelihood method based on the Kimura 2-parameter model. The bootstrap consensus tree inferred from 1000 replicates. The tree is drawn to scale, with branch lengths in the same units as those of the evolutionary distances used to infer the phylogenetic tree.

Phylogenetic analyses (Figure 3) conducted on the alignment of the *rrs* sequences obtained in this study with sequences representative of the diversity of *Leptospira* genus, allowed to identify the three main *Leptospira* groups strongly supported by bootstrap analysis: (a) the pathogenic *Leptospira* spp. group (P1) including LEP_Seq-DOG1-4-5 and the pathogenic reference sequences; (b) the intermediate clade (P2) comprising the LEP_Seq-DOG2 and the LEP_Seq-DOG3 sequence types obtained from this study and the reference sequences of intermediate *Leptospira* species; (c) the saprophytic reference strains of *Leptospira* spp. (S1).

### 3.5. Whole Genome Phylogenetic Analysis of dog4 DNA

**Figure 4:**
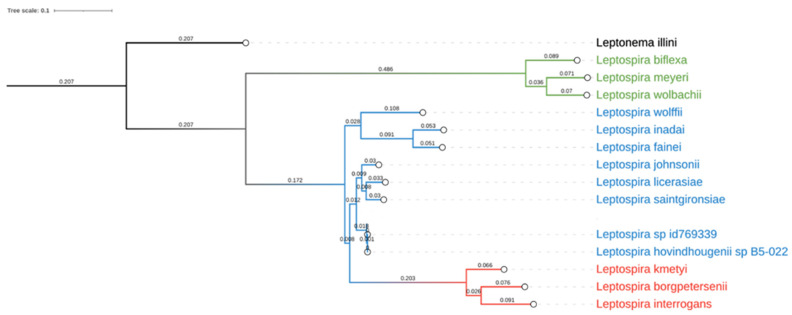
Phylogenetic analysis of *Leptospira* spp. graphical representation of the phylogenetic tree produced by PhyloPh-lAn based on 15 whole genome sequences. *Leptospira* spp. have been differently colored according to their main group: pathogenic in red, intermediates or opportunistic in blue and non-pathogenic in green. *Leptonema illini* was used as an outgroup.

In order to obtain DNA information on the *Leptospira* sequence isolated from dog4 (This Whole Genome Shotgun project has been deposited at DDBJ/ENA/GenBank under the accession JAHZNM000000000), total DNA was sequenced with the Illumina MiSeq technology. A total of 903,510 paired-end 300 bp sequence reads with an average insert size of 355 bp were obtained. After pre-processing, 697,068 read pairs remained, thus corresponding to a predicted genome coverage near to 100X, considering a genome size of 4.3 Mb. 219 scaffolds were included into the final assembly computed by SPAdes [27], showing good metrics (N50 = 148,856 bp, L50 = 10 and largest contig of 373,140 bp). This resulted in an estimated genome size of 4,389,911 bp, with a mean coverage greater than 45X. Lineage analysis revealed a complete overlap with *Spirochaetia* class (239/239 BUSCO marker) [29] and this data was supported also by MetaPhlAn [30], where the specimen was associated with *Leptospira* spp. B5-022 (Accession Number NZ_ANIJ00000000), without presenting any kind of contamination. The proximity with the aforementioned species was confirmed by the phylogenetic analysis, including the new strain of *Leptospira* within the intermediates group (Figure 4).

## 4. Discussion

Dogs are well known reservoir of *Leptospira* serovars and can potentially serve as a source of human infection [33]. Studying of *Leptospira* spp. in dogs, represents a key tool in the understanding of the epidemiology of the disease in the island. The few data on leptospiral infection in canine population available in the Island and the importance to understand the role of dog as possible source of human infections, were the principal reason of this study. In addition, since the clinical presentation of the disease could be nonspecific, and the infection in dogs could be confused with febrile syndrome, hepatic disease, or fever of unknown origin [34], the sequencing of different bacterial strains here detected, allowed us to evolve the knowledge about *Leptospira* serovars circulating in Sardinia.

Serological detection of pathogenic *Leptospira* strains from asymptomatic dogs was recently reported in Sardinia [18]. In the same study, *Leptospira* seropositivity was not correlated with shedding of the bacteria in dog urine and both rt-PCR and urine culture gave negative results. The present study documents the first isolation of pathogenic and intermediate *Leptospira* species from urine cultures collected from five symptomatic dogs. Serological results from this study revealed that dogs of any age, sex as well as those previously immunized are susceptible to leptospirosis infection. Bratislava (serogroup Australis) and Copenhageni (serogroup Icterohaemorrhagiae) were the most frequently serovars detected and it was in agreement with a previous study [18]. Icterohaemorrhagiae, Pomona and Grippotyphosa serovars were also detected, indicating the cocirculation of five different *Leptospira* serovars in the study area. Icterohaemorrhagiae and Copenhageni serovars are the most representative and virulent strains of the Icterohaemorrhagiae serogroup [16,35] and are typically responsible for the majority of severe cases of leptospirosis in humans [1]. In Italy, these serovars have been reported from several domestic mammals, including dogs [36], even if rats of the genus *Rattus* are considered the main reservoir for both serovars [9], suggesting a participation of these hosts in the environmental persistence of the bacterium. Although all dogs tested had been immunized by using the bivalent vaccine, four asymptomatic dogs that received the vaccine less than six months since the blood collection, which reacted with the serovar Icterohaemorrhagiae (with titer ranging from 1:100 to 1:400) that is traditionally included in the composition of the bivalent vaccine. This fact was probably due to a multitude of clinical situations ranging from the high bacterial load to the immunocompromised state of the dogs. In addition, MAT-positive reactions could be related to post-vaccinal cross-reactivity with high antibody titers persisting for few months after vaccination [37]. In contrast to data obtained in the previous study [18], this study showed no MAT positivity for Canicola serovar (the other serovar present in vaccine formulation), confirming the health strategy of vaccines to prevent infection as well as induce protective immunity against leptospirosis. Coinfections with more than one *Leptospira* serovars, due to a MAT cross reaction between antigens of different serovars, or a mixed infection were also detected in this study (Table 2 and Table 3). In the symptomatic dog2 and dog5 that presented a form of acute leptospirosis, three and four different serovars were detected by MAT, respectively. In particular, dog5 presented higher titers for Bratislava (1:3200) and Grippotyphosa (1:800) serovars, indicative of high bacterial loads responsible for the disease manifestation. In dog2 a triple coinfection with Pomona, Bratislava, and Copenhageni serovars at antibody titers of 1:200, 1:400, and 1:800, was also detected (Table 2). We could postulate that clinical symptoms in these dogs may have been exacerbated by the simultaneous acquisition of the different strains that led to dog deaths. However, further studies are needed to support these serological findings by culture isolation of these multiple isolates. In three dogs (the symptomatic dog1 and dog3 plus the asymptomatic dog7), a 3-fold increase in antibody titers confirmed a recent infection and the failure of the antimicrobial therapy. No seroconversion was observed in the other dogs probably due to initiation of antibiotic therapy that allowed to avoid more severe complications (Table 3). Serological monitoring should be performed every 2–4 weeks in cases of suspected leptospiral infection since single high titers (≥1:800) do not confirm a diagnosis of leptospirosis [38]. Only 7/17 urine samples resulted positive after multiplex real time PCR (see Table 2). It could be related to the small number of leptospires in the matrix (<4.9 × 10^4^ cell/mL), and also to PCR inhibitors that could be present in urine. In addition, the first isolation of *Leptospira* strains here successfully obtained from four urine samples and one kidney allowed us to postulate that bacterial loads in symptomatic dogs could be significantly higher than in asymptomatic dogs. However, further experimental studies are warranted in order to clarify this hypothesis. Furthermore, we failed to isolate *Leptospira* from urine of asymptomatic dogs, indicating that culture isolation has multiple limitations and this could not be used to make a definitive diagnosis [39]. Negative results from urine of asymptomatic dogs should be taken with more attention since, in case of leptospiremia, the urinary shedding could be delayed and intermittent [18]. Asymptomatic dogs could shed *Leptospira* spp. via urine and contaminate the environment playing a role as carries of leptospires in this area. In addition to these results, the isolation of one *Leptospira* strains from the intermediate cluster was likely the most interesting finding of this work. Using the 16S rRNA PCR method, pathogenic *Leptospira interrogans* and intermediate *Leptospira* spp. were found distributed within the study area. However, DNA sequencing of *rrs* sequences highlighted that this target gene should not be used alone, but integrated with the sequences of other conserved marker genes. The *secY* gene was further used in this study as it has been widely used for diagnosis of animal leptospirosis, presenting a good discriminatory power, and sequence analysis of this gene allows identification at species level. The analysis of *secY* on urine samples did not give results after amplification and the whole genome of the *Leptospira* isolate was sequenced. Genotyping analysis allowed us to evolve the knowledge about *Leptospira* serogroup circulating in the Island. We generated four sequences from seven MLST housekeeping genes belonging to three different sequence types. In particular, the ST198 and ST24 were found in kidney and urine cultured from dog1 and dog5, respectively. Although the more severe clinical signs are associated with serogroup Icterohaemorrhagiae in dogs, data regarding clinical manifestations of infection caused by the serogroup Australis are limited. Sequence type 198 was already described in hedgehogs from the study area [18], Northern Italy, and several other European countries [40]. Thus, the indirect transmission of the pathogen through a contaminated shared environment has been postulated in the case of dog1. Clinically, the dog showed an initial clinical presentation (fever, anorexia, and weight lose) that might not be recognized by physicians and it may be misdiagnosed as it mimics many other diseases severe. Regarding the prognosis, we correlated dog deaths with the presence of serogroup Australis which to date has been related to severe life-threatening clinical presentation as described recently in dogs from Asia [41]. Further studies are needed to evaluate the correlation of severe clinical presentation of dogs with pathogenicity related to serogroup Australis. Moreover, this work reports the first whole genome sequencing of an intermediate *Leptospira* species isolated from the urine of the symptomatic dog3. Mild symptomatic forms were associated to this dog (fever and anorexia) and it was in agreement with other reports in which less severe symptoms caused by intermediate strains in comparison to those caused by the pathogenic *Leptospira* have been described in humans. Severe symptoms have been associated only with the intermediate cluster species *L. broomii* [42]. Although the strain here detected was previously isolated from a rat in Denmark, no information regarding the virulence as well as the clinical symptoms caused by this strain in hosts are currently known and further experimental studies are warranted.

## 5. Conclusions

The identification of urinary shedding of leptospires in symptomatic companion dogs highlights preventive veterinary measures which should be taken to prevent dissemination of the leptospires in the environment and between animals, including humans. Thus, monitoring of leptospiral infection in dogs can be helpful as marker to predict pathogen transmission in human, and monitor changes in pathogen prevalence or incidence. Moreover, information on the intermediate strain here isolated must be implemented in further studies in order to provide knowledge on bacterium’s virulence as well as on its ability to induce clinic manifestations in infected hosts.

## Figures and Tables

**Table 1:** Dogs enrolled in this study classified on the basis of health status, origin place, sex and age.

Health State	Total Dogs	N. Dogs (Place)	Sex		Age	
			♂	♀	≤2	>2
Asymptomatic	150	108 (Sassari) 18 (Alghero) 15 (Porto Torres) 6 (Sorso) 3 (Ploaghe)	78	72	62	88
Symptomatic	25	20 (Sassari) 3 (Alghero) 2 (Porto Torres)	15	10	8	17
Total	175		93	82	70	105

**Table 2:** Data on sex, age, habitat, clinical symptoms, rt-PCR and, titers for some serovars of the 17 dogs that tested positive for MAT at 1st sampling.

Dog	Gender	Age (Months)	Habitat	Vaccination Status	Clinical Signs	MAT Titers (1st Sampling)					rt-PCR (*lipL32* Gene)
						Brat.	Grippo.	Pomona	Ictero.	Copen.	
1	Male	15	Urban	<6 months	Fever, anorexia, weight lose	1:400	1:400	1:400	-	1:200	Pos
2	Male	18	Urban	<6 months	Icterus, anorexia, weight loss	1:400	-	1:200	-	1:800	Pos
3	Female	18	Urban	<6 months	Fever, anorexia	1:400	-	-	-	-	Pos
4	Female	28	Urban	<6 months	Prostration, vomiting	1:800	-	1:200	-	1:1600	Pos
5	Male	30	Rural	>6 months	Gingival lesions jaundice, haemorrhagic disorders, hyperoxia	1:3200	1:800	1:100	-	1:200	Pos
6	Male	40	Rural	>6 months	None	1:400	-	-	-	-	Pos
7	Male	48	Rural	>6 months	None	-	-	-	1:400	1:400	Pos
8	Male	32	Rural	>6 months	None	1:200	-	-	-	-	Neg
9	Female	36	Rural	>6 months	None	-	-	-	-	1:100	Neg
10	Female	32	Rural	>6 months	None	1:200	-	-	-	-	Neg
11	Female	18	Urban	<6 months	None	-	1:100	-	-	-	Neg
12	Female	30	Urban	>6 months	None	-	-	-	1:200	-	Neg
13	Female	26	Urban	>6 months	None	1:200	-	-	-	-	Neg
14	Female	20	Urban	>6 months	None	1:200	-	-	-	-	Neg
15	Female	30	Urban	<6 months	None	1:100	-	-	-	1:200	Neg
16	Male	18	Urban	>6 months	None	-	-	-	1:100	-	Neg
17	Male	48	Urban	>6 months	None	-	-	-	1:100	-	Neg

**Table 3:** List of resulted MAT positive after the first sampling and titers of leptospiral antibodies from seroconverted dogs after two weeks (2nd sampling).

N° Dog	Serological Titer—Sampling 1st/2nd									
	Brat.		Pomona		Copen.		Ictero.		Grippo.	
	1st	2nd	1st	2nd	1st	2nd	1st	2nd	1st	2nd
1	1:400	1:400	1:400	<1:100	1:200	1:1600			1:400	<1:100
2	1:400	1:800	1:200	1:200	1:800	1:800				
3	1:400	1:1600								
4	1:800	1:100	1:200	<1:100	1:1600	1:100				
5	1:3200		1:100		1:200				1:800	
6	1:400	1:400								
7					1:400	1:400	1:400	1:1600		
8	1:200	<1:100								
9					1:100	1:100				
10	1:200	1:100								
11									1:100	1:100
12							1:200	1:100		
13	1:200	1:100								
14	1:200	<1:100								
15	1:100	1:100			1:200	1:200				
16							1:100	1:100		
17							1:100	1:200		

**Table 4:** Results *rrs* and *secY* PCR, and MLST performed on *Leptospira* spp. isolated from urine and kidney of positive dogs in Table 2.

Dog	Source	Characterization of *Leptospira* Isolates		
		*rrs* PCR	*secY* PCR	MLST (species)
1	kidney	*L. interrogans*	*L. interrogans*	ST 198 (*L. interrogans* Australis)
2	urine	*L. inadai*	*L. interrogans*	ST 17 (*L. interrogans* Copenhageni)
3	urine	*L. saintgironsiae*	No amplification	No amplification
4	urine	*L. interrogans*	*L. interrogans*	ST 17 (*L. interrogans* Copenhageni)
5	urine	*L. interrogans*	*L. interrogans*	ST 24 (*L. interrogans* Bratislava)

## Data Availability

Whole Genome Shotgun project has been deposited at DDBJ/ENA/GenBank under the ac-cession JAHZNM000000000.
